# DVT Surveillance Program in the ICU: Analysis of Cost-Effectiveness

**DOI:** 10.1371/journal.pone.0106793

**Published:** 2014-09-30

**Authors:** Ajai K. Malhotra, Stephanie R. Goldberg, Laura McLay, Nancy R. Martin, Luke G. Wolfe, Mark M. Levy, Vishal Khiatani, Todd C. Borchers, Therese M. Duane, Michel B. Aboutanos, Rao R. Ivatury

**Affiliations:** 1 Department of Surgery, Virginia Commonwealth University, Richmond, Virginia, United States of America; 2 Department of Biostatistics, Virginia Commonwealth University, Richmond, Virginia, United States of America; The National Institute for Health Innovation, New Zealand

## Abstract

**Background:**

Venous Thrombo-embolism (VTE – Deep venous thrombosis (DVT) and/or pulmonary embolism (PE) – in traumatized patients causes significant morbidity and mortality. The current study evaluates the effectiveness of DVT surveillance in reducing PE, and performs a cost-effectiveness analysis.

**Methods:**

All traumatized patients admitted to the adult ICU underwent twice weekly DVT surveillance by bilateral lower extremity venous Duplex examination (48-month surveillance period – SP). The rates of DVT and PE were recorded and compared to the rates observed in the 36-month pre-surveillance period (PSP). All patients in both periods received mechanical and pharmacologic prophylaxis unless contraindicated. Total costs – diagnostic, therapeutic and surveillance – for both periods were recorded and the incremental cost for each Quality Adjusted Life Year (QALY) gained was calculated.

**Results:**

4234 patients were eligible (PSP – 1422 and SP – 2812). Rate of DVT in SP (2.8%) was significantly higher than in PSP (1.3%) – p<0.05, and rate of PE in SP (0.7%) was significantly lower than that in PSP (1.5%) – p<0.05. Logistic regression demonstrated that surveillance was an independent predictor of increased DVT detection (OR: 2.53 – CI: 1.462–4.378) and decreased PE incidence (OR: 0.487 – CI: 0.262–0.904). The incremental cost was $509,091/life saved in the base case, translating to $29,102/QALY gained. A sensitivity analysis over four of the parameters used in the model indicated that the incremental cost ranged from $18,661 to $48,821/QALY gained.

**Conclusions:**

Surveillance of traumatized ICU patients increases DVT detection and reduces PE incidence. Costs in terms of QALY gained compares favorably with other interventions accepted by society.

## Introduction

Venous thrombo-embolism (VTE), encompassing deep vein thrombosis (DVT) and pulmonary embolism (PE) causes significant morbidity and mortality among traumatized patients. Without prophylaxis, the incidence of DVT is estimated to be 60% [1] dropping to 6–21% with adequate mechanical and/or pharmacologic prophylaxis. [2]–[8] In view of this, prophylaxis against VTE is standard of care for traumatized patients at high risk of VTE. With or without prophylaxis, patients that do develop DVT are at risk of short term complications (limb threatening phlegmasia, and life threatening PE) and long term complications (post-phlebetic limb, and pulmonary hypertension). Thus the total burden of VTE in traumatized patients is significant both in terms of cost and suffering.

Despite adequate prophylaxis, [9] DVT does occur and can be silent with the first manifestation being a PE that is fatal in 5–10%. [10], [11] Surveillance screening for DVT in high risk patients may be an effective strategy to diagnose and treat DVT and thus reduce both short and long term morbidity and mortality. The current study evaluates the cost effectiveness of such a surveillance program on traumatized patients at high risk of VTE, with a particular focus on the quality adjusted life years (QALY) gained from any reduction in PE.

## Materials and Methodology

The study was performed at the level-I trauma center at the Virginia Commonwealth University (VCU) Medical Center. The trauma center is a state designated and American College of Surgeons verified level-I center in central Virginia serving a population of two million, with annual trauma admissions of >4,000. The study was approved by the Institutional Review Board (IRB) of VCU. The study was exempt from informed consent since 1. all the data was already gathered in the registry of the trauma center prior to the conception of the study and 2. data from the registry was de-identified.

All adult traumatized patients admitted to an intensive care unit (ICU) were included. During the 36-month pre-surveillance period, patients received mechanical prophylaxis (thigh-high sequential compression device) *and* pharmacologic prophylaxis [low molecular weight (LMW) heparin], unless contraindicated. Prophylaxis was initiated within 24 hours of admission. In the year 2000 the institution adopted the policy of initiating un-fractionated heparin for all head injured patients within 24 hours of admission unless the patient had an expanding head bleed and this policy was continued throughout the study period. The prophylaxis protocol did not change over the time course of the study, and the non-compliance rate was <10%. During the 48-month surveillance period, in addition to pharmacologic prophylaxis, patients underwent twice weekly DVT surveillance of both lower extremities by bedside venous Duplex examination, till discharged from the unit. Surveillance was performed on the lower extremities since 90% of the PEs originate in the lower extremity, [12] and the focus of the study was to examine the reduction in PEs by early detection and prompt therapy of DVT. Patients diagnosed with clinically significant (popliteal vein or higher) DVT were initially treated with therapeutic heparin (unfractionated or LMW) and later either maintained on LMW heparin or changed to oral anti-coagulation. In instances where therapeutic anti-coagulation was contra-indicated, or there was progression of VTE despite adequate anti-coagulation, inferior vena-caval filter was inserted to prevent PE. Anti-coagulant therapy was continued for at least six months. DVTs that involved only the infra-popliteal calf veins were not treated, however prophylaxis and surveillance was continued.

Each incidence of DVT and PE was recorded. Cost determinations were based on the following: Diagnosis/surveillance for DVT = $200/duplex study; diagnosis of PE by contrast enhanced computed tomography (CECT) of chest  = $500; VTE therapy cost  = $6,000. [13] During PSP, total cost was calculated by adding the diagnostic cost of each incidence of DVT (single venous duplex examination) and PE (single CECT of chest) and the therapeutic cost of all VTE requiring therapy. For SP surveillance costs were calculated by multiplying the cost of a single Duplex study by the average number of studies per ICU patient under surveillance. The average number of studies per patient was determined by dividing the average ICU length of stay in days by 3.5 since surveillance studies were performed twice in one week. To the surveillance cost was added the diagnostic cost of each incidence of PE, and the therapeutic cost of all VTE requiring therapy. For cost calculations in SP, it was assumed that all DVT detected was via surveillance, hence there are no separate diagnostic cost for DVT.

### Statistical and Cost benefit analysis

Uni-variate comparisons were performed using the appropriate tests – Chi-square, with Fisher's exact correction where required, for discrete variables, and Student's t-test for parametric and Wilcoxson rank sum test for non-parametric continuous variables. Regression analysis was performed to evaluate the independent predictors of DVT and PE including the role of surveillance. Significance was set at p<0.05. For cost benefit analysis, the total cost of the program was calculated as detailed above. This cost was used to calculate the cost per life saved if the program led to a decrease in number of PEs. All costs were computed in US dollars. All calculations were performed using Statview software (SAS Institute Inc Cary NC, USA). All continuous data is presented as Mean±Standard Error of Mean (SEM).

## Results

There were 4234 trauma patients admitted to the ICU during the 84-month study period (Table 1). The overall characteristics of the population were similar to most trauma populations with a relatively young age, and male preponderance. Of these, 1422 were during PSP and 2812 during SP.

**Table 1:** Patient populations in the two periods of study.

	PSP (n = 1422)	SP (n = 2812)
**Age (years)**	40.76±0.50	40.69±0.36
**Gender (% male:female)**	72∶28	73∶27
**Mechanism (% blunt:penetrating)** *	81∶19	84∶16
**ISS** *	19.37±0.33	20.89±0.25
**RTS**	6.95±0.05	6.89±0.04
**AIS**		
** Head** *	2.93±0.04	3.14±0.03
** Face** *	1.42±0.03	1.95±0.03
** Chest** *	3.05±0.04	3.29±0.02
** Abdomen**	2.74±0.04	2.66±0.03
** Extremity** *	2.28±0.03	2.55±0.02
** External** *	1.12±0.04	1.04±0.01
**Inferior Vena Caval filter** *	34 (2%)	28 (1%)
**Mortality**	129 (9.1%)	250 (8.9%)

*p<0.05: PSP vs SP. PSP: pre-surveillance period, SP: surveillance period, ISS: injury severity score, RTS: revised trauma score, AIS: Abbreviated Injury Scale.

### VTE

There were 139 incidents of VTE (DVT-96; PE-43) among 129 patients. The characteristics of patients that developed VTE and those that did not are presented in Table 2. Patients developing VTE were older with a higher injury severity score (ISS) – p<0.05 for both. VTE was more common in males, and those that suffered blunt trauma, though these differences were not statistically significant – p>0.05 for both. VTE was also more common in patients sustaining injuries to face, chest and extremity as evidenced by the higher abbreviated injury scale (AIS) for these body regions – p<0.05 for all. Older age and higher ISS was observed in the DVT patients (p<0.05), but only ISS was significantly higher in the PE patients (p<0.05). Like VTE in general, both DVT and PE patients demonstrated a male preponderance and more blunt mechanism, however these differences were not statistically significant (p>0.05). Among the 96 DVTs, 18 were in PSP, and of these nine (50%) were clinically significant, requiring therapy as per protocol. The remaining 78 DVTs were in SP, and of these 47 (60%) were clinically significant. Five of these 47 were in patients in whom surveillance had previously detected calf vein DVT, and with continued surveillance the DVT was observed to progress. Of the 43 PEs, 22 were in PSP and 21 in SP.

**Table 2:** Details and outcomes of patients developing venous thrombo-embolism (VTE) or not.

	No VTE (n = 4105)	VTE (n = 129)	DVT (n = 96)	PE (n = 43)
Age (years)*^#^	40.52±0.3	47.11±1.62	48.51±1.99	42.90±2.30
**Gender (% male:female)**	72∶28	78∶22	78∶22	84∶16
**Mechanism (%blunt:penetrating)**	83∶17	87∶13	85∶15	93∶7
ISS*^#∧^	20.09±0.20	28.46±1.13	29.67±0.20	25.66±1.81
**RTS**	6.91±0.03	6.72±0.18	6.82±0.19	6.54±0.42
**AIS**				
** Head**	3.07±0.02	3.22±0.13	3.07±0.02	3.23±0.26
Face*	1.70±0.02	1.92±0.12	1.86±0.14	2.00±0.22
Chest*^#^	3.21±0.02	3.44±0.10	3.60±0.11	3.19±0.19
** Abdomen**	2.69±0.02	2.78±0.12	2.79±0.13	2.76±0.25
Extremity*^#^	2.44±0.02	2.64±0.08	2.70±0.08	2.50±0.14
** External**	1.04±0.01	1.06±0.06	1.08±0.08	1.00±0.00
ICULOS (days)*^#∧^	5.59±0.14	20.15±1.67	22.03±2.02	13.19±2.12
HLOS (days)*^#∧^	11.78±0.23	35.88±2.76	36.90±3.09	30.79±4.87
Mortality*^#∧^	356 (8.3%)	23 (17.8%)	16 (16.7%)	8 (18.6%)

Among those developing VTE, details of those developing deep vein thrombosis (DVT), or pulmonary embolism (PE). p<0.05: *No VTE vs VTE, ^#^No DVT vs DVT, ^∧^No PE vs PE. ISS: injury severity score, RTS: revised trauma score, AIS: abbreviated injury scale, ICULOS: Intensive Care Unit Length of Stay, HLOS: Hospital Length of Stay.

**Figure 1:**
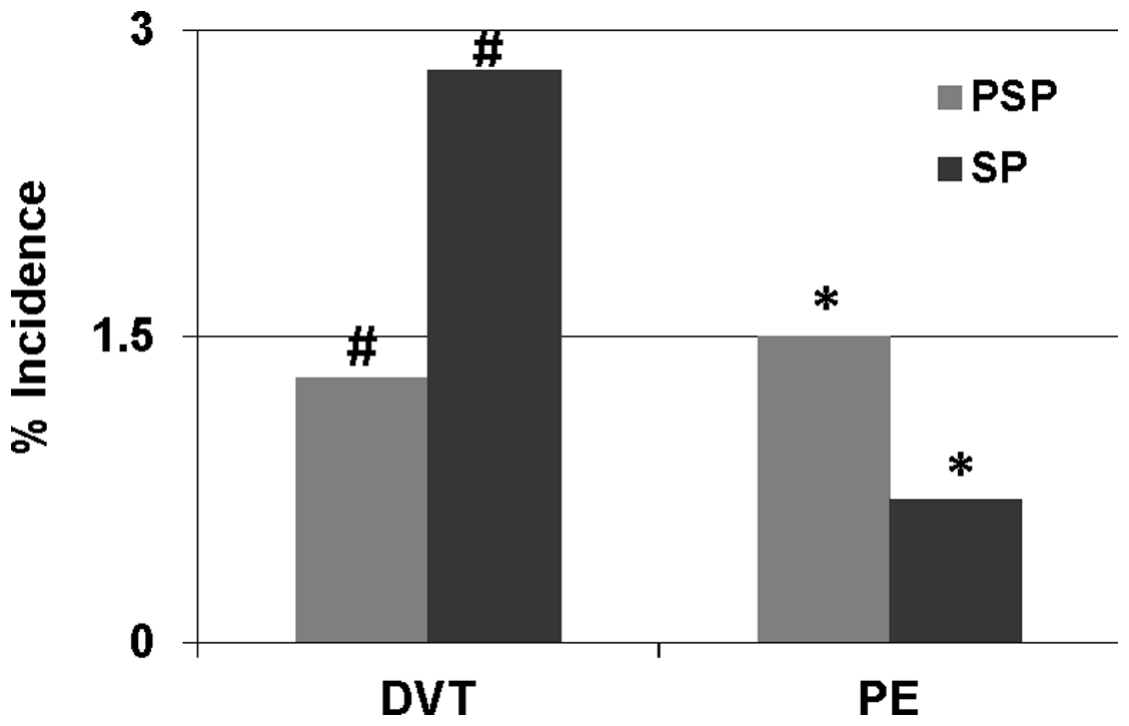
Figure showing the rates of deep vein thrombosis (DVT) and pulmonary embolism (PE) during the pre-surveillance period (PSP: 2001–03) and the surveillance period (SP: 2004–07). #p<0.05 – DVT (PSP vs SP); *p<0.05 – PE (PSP vs SP).

The rate of DVT increased while the rate of PE decreased between PSP and SP. The overall rate of DVT was 2.2% (96/4234). The rate during PSP was 1.3% (18/1422), and with surveillance, the rate increased by 115% to 2.8% (78/2812). The overall rate of PE was 1% (43/4234). The rate of PE during SP (0.7% – 21/2812) was 50% lower than in PSP (1.5% – 22/1422). The differences in the rates of both DVT and PE during PSP vs SP, were statistically significant – p<0.05 for both (Fig. 1). Since there were some differences in the patient population and injury characteristics between the two periods (Table 1), a logistic regression model was constructed to identify independent predictors of DVT and PE (Table 3). Among the patient and injury characteristics, age, ISS and AIS of extremity were independently predictive of DVT, and AIS of chest and extremity were independently predictive of PE. After controlling for all variables identified, surveillance was an independent predictor of increased DVT detection (OR 2.53 with 95% CI of 1.462–4.378) and of decreased PE incidence (OR 0.487 with 95% CI of 0.262–0.904).

**Table 3:** Results of logistic regression showing the independent predictors of Deep Vein Thrombosis (DVT) and Pulmonary Embolism (PE).

	DVT		PE	
	OR	95% CI	OR	95%CI
**Age**	1.022	1.011–1.032	-	-
**ISS**	1.039	1.023–1.055	-	-
**AIS Chest**	-	-	1.318	1.094–1.587
**AIS Extremity**	1.379	1.168–1.627	1.297	1.025–1.641

OR: odds ratio, CI: confidence interval, ISS: injury severity score, AIS: abbreviated injury scale.

### Outcomes

Occurrence of VTE significantly worsened outcomes in terms of increased mortality and longer lengths of stay in the ICU and the hospital – p<0.05 for all (Table 2). These differences remained when the VTE group was separated into those with DVT and those with PE (Table 2). Overall mortality in PSP was 9.1% and in SP was 8.9% (p>0.05).

### Data assumptions, and Cost calculations

To compute the incremental cost/QALY gained, the following assumptions were made:

1. Attention was restricted to men aged 50 and older to be consistent with the cohort studied. Survival for men up to age 99 was computed using CDC data. [14]
2. The base case health utility for all patients was 0.9, which is consistent with publicly reported values associated with DVT. [15] For sensitivity analysis, this was varied from 0.8–1.0.
3. All health effects were discounted at 3% which is also standard in terms of reduction in quality of life with age. For sensitivity analysis the discount rate was varied from 0–5%.
4. To calculate cost per life saved, it was assumed that the main difference between SP and PSP was a reduction in PEs. The difference in PE incidence rate between PSP and SP was computed using the observations from the study – an absolute reduction of 0.8% (1.5% in PSP to 0.7% in SP). For sensitivity analysis 95% confidence interval for the reduction in the PE rate was utilized. This, using the normal approximation to the binomial distribution, is 0.2%–1.4%.
5. The PE fatality rate for the base case was assumed to be 7.5%, and a range from 5–10% was considered for sensitivity analysis. [10], [11]
6. Those who survive PE suffer from a short-term loss in 0.0115 QALYs due to short-term hospitalization. [16]

The total VTE related patient care cost during PSP (diagnostic and therapeutic) was $194,600/1422 patients – $136,850/1000 patients and during SP (diagnostic, therapeutic and surveillance) was $1,244,100/2812 patients – $442,425/1000 patients. Thus, the total additional cost of the surveillance program was $305,575/1000 patients. During this period the PE rate decreased from 1.5% to 0.7% or, eight PEs were prevented per 1000 patients placed under surveillance. Assuming a base case PE fatality rate of 7.5%, it is necessary to prevent 13.33 PEs in order to save one life. This translates to 1666 patients placed under surveillance at a cost of $509,091 per life saved.

The incremental cost per QALY saved was computed as follows. Future expected life years were computed using CDC mortality data for men aged 50 and over to be consistent with the cohort studied. [14] Given these assumptions, patients not experiencing a PE fatality would live an additional 30.31 expected life years, which translates to an additional 17.49 quality-adjusted/discounted life years with a health state utility of 0.9. This yielded an incremental cost of $29,102/QALY gained for the base case.

## Discussion

VTE is a major problem for high risk trauma patients. A large body of literature has established that trauma patients are at higher risk of developing DVT that can lead to mortality from PE, and both short and long term morbidity. Without prophylaxis the incidence of DVT in trauma patients has been reported to be as high as 60%. [1] This has resulted in some form of prophylaxis becoming a standard of care for all high risk trauma patients. [17]

Despite prophylaxis, DVT occurs in 6–12% of trauma patients. [8] Oftentimes the DVT itself is silent, with the first sign of VTE being a pulmonary embolism that is fatal in 5–10% of instances. [10], [11] Routine surveillance for DVT maybe a method to diagnose DVT at an asymptomatic stage, with the hope that by therapy and/or placement of a protective inferior vena-caval filter, a large hemodynamically compromising or fatal PE will be prevented. The value and cost-effectiveness of this approach is debated. In an early prospective study, Piotrowski et al reported on 343 high-risk trauma patients placed on DVT surveillance and concluded that since with prophylaxis the risk of DVT was low, the cost of detecting a single DVT by surveillance was extremely high. [18] They did however suggest that routine DVT surveillance may reduce the risk of PE. [18] In a retrospective study Meyer et al reported on traumatized ICU patients and concluded that at US$ 6688/DVT detected, the cost was too high to recommend routine surveillance, but it should be considered in patients at the highest risk. [19] Major et al reported on 726 patients admitted to the surgical ICU (majority due to trauma) of which some underwent routine DVT surveillance and concluded that with adequate prophylaxis the incidence of both DVT and PE were low and hence, surveillance was not indicated. [20] Similar doubts regarding the utility and cost-effectiveness of surveillance have been reported by others. [21]–[24] Based on the results of these studies, the latest edition (9^th^) of the American College of Chest Physicians (ACCP) evidenced based guidelines on DVT prevention state that the benefit of DVT surveillance by duplex sonography in reducing the incidence of PE and mortality is unclear. Additionally, since up to 30% of screen positives maybe false, [25] there is potential harm in that the falsely positive patients may get unnecessary anticoagulation therapy with its attendant risks. [9] On the other hand, Napolitano et al reported on 458 high-risk traumatized patients that underwent routine surveillance, and found a significant incidence of asymptomatic DVT concluding that for the high-risk trauma patient, surveillance is justified. [26] Adams et al, in a large recent study involving>4,000 traumatized patients of whom 982 high-risk patients were placed on DVT surveillance, reported that 86% of the lower extremity DVTs were clinically silent and detected by surveillance. They concluded that surveillance was justified in this patient population. [27] In light of these conflicting conclusions, the CDC convened a panel of experts to examine and advise about the utility of DVT surveillance in hospitalized patients. The panel recommended that surveillance can provide robust data on the epidemiology of VTE and effectiveness of prevention programs however more data was needed regarding the utility of surveillance itself in improving patient outcomes. [28] The current study reports on a surveillance program at a busy level-I trauma center, and aims at performing a detailed cost effectiveness analysis of such a program. In the current economic and healthcare environment, merely demonstrating a medical benefit may no longer be enough to support an intervention. The direct cost and the resultant benefit has to be at a level that society is willing and able to afford. Past DVT surveillance studies have focused on establishing the incidence and risk factors for VTE, however, to the best of our knowledge, this is the first report of QALY based cost effectiveness analysis of such a program in an acute care setting.

VTE was seen more often in older patients with a higher ISS and those with injuries to extremity and the chest regions. This is similar to what has been reported in many studies. [1], [6], [7], [18], [27] Unlike other studies, the current study failed to demonstrate a higher incidence of VTE in head injured patients. This may be related to the very aggressive prophylaxis program instituted at our institution in 2000 where all head injured patients received unfractionated heparin within 24 hours of admission, unless the repeat CT demonstrated expansion of intra-cranial hemorrhage. Surveillance resulted in higher numbers of DVTs being diagnosed and treated, and at the same time, lowering the incidence of PE (Fig. 1). Regression analysis demonstrated surveillance to be an independent predictor of increased DVT detection and decreased PE incidence. We believe that the increase in the numbers of DVT diagnosed, and treated directly resulted in a lower incidence of PE. While clearly effective in decreasing the number of PEs, we did not see a significant decrease in mortality. The total incidence of PE was 43 or 1% of the total population. This coupled with the known PE fatality rate of 5–10% means that a very large number of patients will be required to demonstrate a significant decrease in the PE related mortality by such a surveillance program.

In the current study, the incremental cost per life saved was $509,091. In comparison, post-exposure prophylaxis against rabies is estimated to cost>$500,000 per life saved. [29] In the non-disease arena, in 2002, The National Highway Traffic Safety Administration (NHTSA) evaluated the cost benefit of all mandated Federal Motor Vehicle Safety Standards (FMVSS). The report concluded that these standards helped save 20,851 lives in 2002 at a cost of $544,482 per life saved. [30] There was only one study that directly evaluated cost-effectiveness of DVT surveillance. In that 1996 study on traumatic and non-traumatic brain injured patients, that underwent a single screening duplex examination at the time of admission to the rehabilitation unit, the cost per life saved was $129, 527. [31] The cost in the current study is much higher. The reasons for that are likely twofold: first is medical care cost inflation, with the current study basing all costs of 2004 estimates as compared to the cited study on 1996 estimates, and secondly, the current study bases costs on twice weekly surveillance examinations, while the brain injury study had a single screening examination at the time of admission only. Cost per life saved however, is a very crude measure of cost benefit evaluation since the method treats each life saved as equivalent irrespective of the future life expectancy at the age death was prevented.

**Figure 2:**
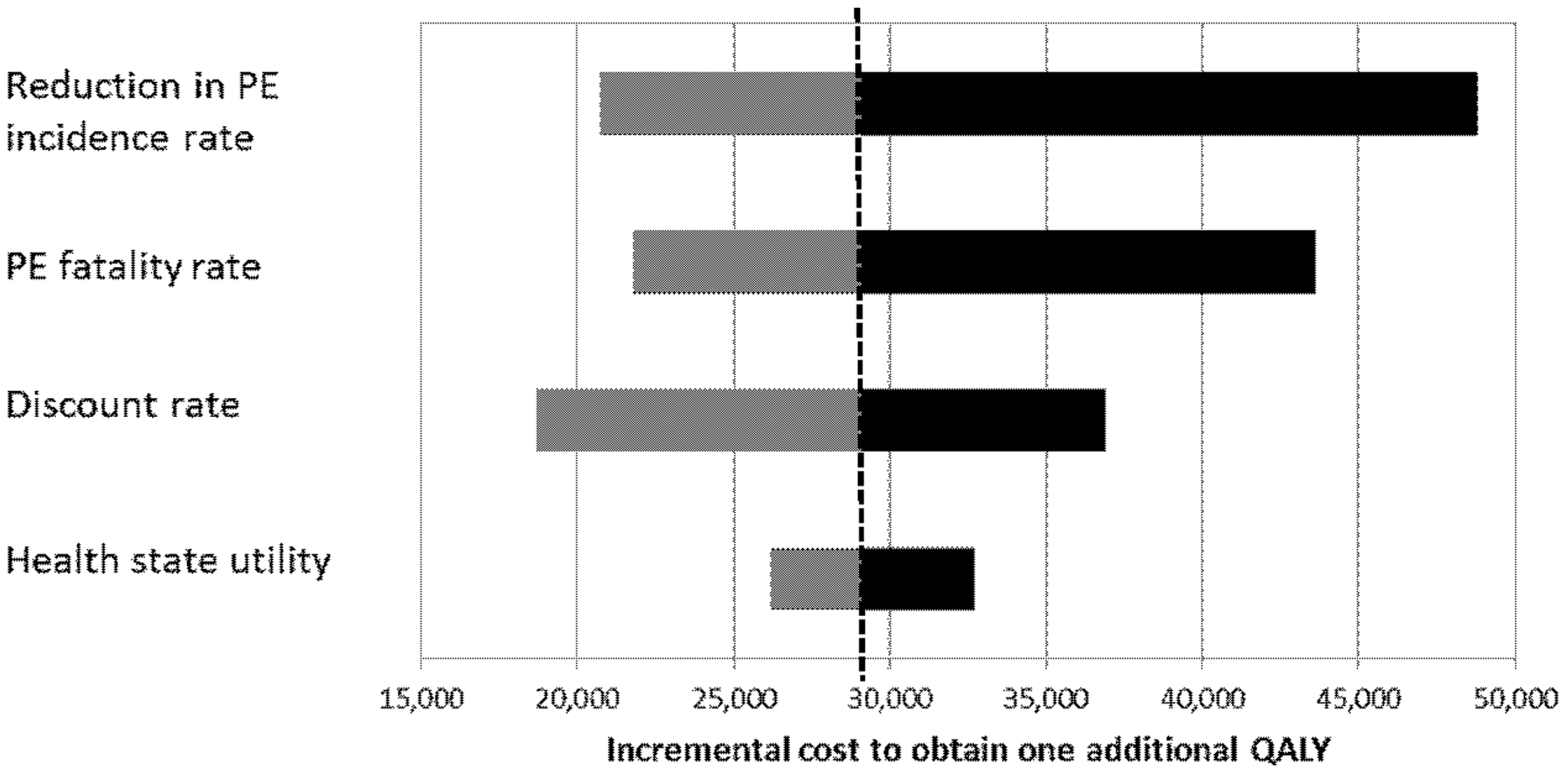
Figure showing a sensitivity analysis on the incremental cost to obtain one additional QALY. The bars represent upper and lower bounds on the incremental cost relative to the base case of $29,102 (dashed line) with respect to the bounds on each parameter value. In this figure, the discount rate ranges from 0% to 5%, the difference in the PE incidence rate between PSP and SP ranges from 0.2% to 1.4%, the PE fatality rate ranges from 5% to 10%, and the health state utility ranges from 0.8 to 1.0.

**Figure 3:**
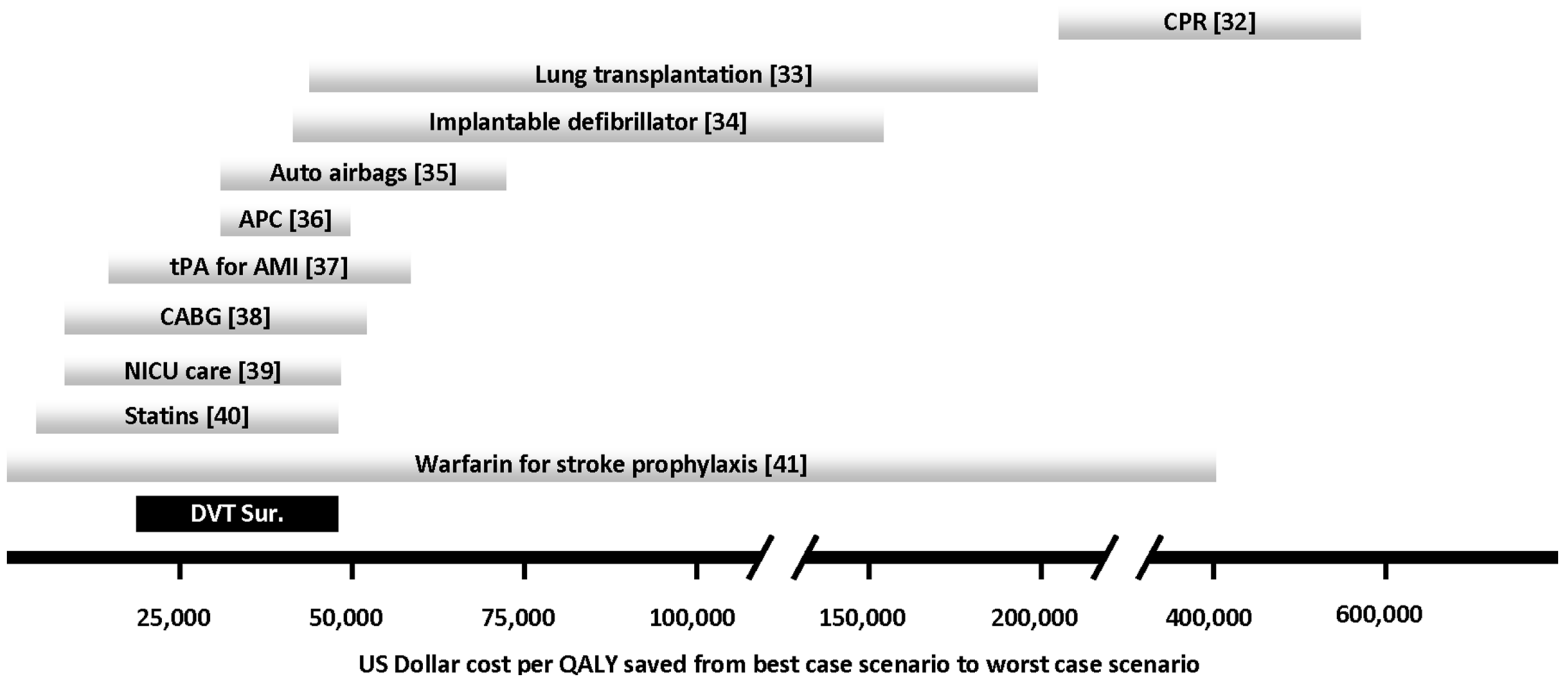
Incremental dollar cost per quality adjusted life year (QALY) saved for selected interventions as compared to the cost of deep venous thrombosis surveillance (DVT Sur.). The bars represent range of cost from the best case scenario (most cost effective) to the worst case scenario (least cost effective) for each intervention. CPR: cardio-pulmonary resuscitation, APC: activated protein C for sepsis, tPA for AMI: tissue plasminogen activator for acute myocardial infarction, CABG: coronary artery bypass grafting, NICU: neonatal intensive care unit. The bracketed number represents the reference the data is derived from.

In the current study, to account for the difference in the quantity and quality of life saved, the incremental cost per QALY was computed at $29,102/QALY gained. To further analyze this value, a uni-variate sensitivity analysis was performed over four of the parameters used in the cost-effectiveness analysis: the discount rate (base case 3% with range of 0%–5%), the difference in the PE incidence rate between PSP and SP (base case 0.8% with range of 0.2%–1.4%), the PE fatality rate (base case 7.5% with range of 5%–10%), and the health state utility (base case 0.9 with range of 0.8–1.0). The resulting incremental costs per QALY saved in the uni-variate sensitivity analysis are illustrated in Fig. 2. The results show that the incremental costs are relatively insensitive to the health state utility ($26,191 – $32,739). The results are somewhat more sensitive to the PE fatality rate, the reduction in the PE rate, and the discount rate, which result in incremental costs per QALY saved of $21,829 – $43,652, $20,729 – $48,821, and $18,661 – $36,905, respectively. The sensitivity analysis results suggests that in all scenarios, the incremental costs compares well with a number of other life saving interventions that are accepted by society in the medical and non-medical arenas as shown in Fig. 3 (compiled from references [32]–[41].

The question as to how much a society is able and willing to afford for each QALY saved is a difficult one. The answer depends upon the societal values and the economic status of an individual society. In the United States a figure of $50,000 per QALY saved is oftentimes utilized to make decisions about resource allocation. [42] Recently that figure has been questioned and it is argued that based on current societal expectations, the figure should be significantly higher. [30] However, even by the traditional model of $50,000 per QALY saved as cost effective, the DVT surveillance program is cost effective under nearly all of the scenarios considered.

There are some limitations to this study. Firstly, it is a retrospective analysis. Due to this, we cannot be sure of the compliance with surveillance. Second, it is not powered to demonstrate a difference in mortality. Since with prophylaxis, the incidence of PE is low (∼1%) and the mortality from PE is <10%, only a very large multi-institutional study could have enough power to demonstrate a difference in PE related mortality. Third, even though the prophylaxis protocol has not changed over the study period, some other subtle changes may have occurred that are missed in a study spanning seven years. Finally, probably the most significant limitation of the study is that while the increased DVT detection and decreased PE incidence were directly observed (measured) and are likely due to surveillance, all the cost calculations leading to the dollar cost/QALY gained, are extrapolations of the data and represent *potential* estimates per QALY gained, if the observations of this study true. As mentioned above, the study is not large enough to demonstrate an actual difference in mortality that maybe attributable to surveillance. Despite these limitations, the study demonstrates that DVT surveillance among traumatized ICU patients increases DVT detection, decreases PE incidence and is cost-effective.

## Conclusions

A DVT surveillance program for trauma patients admitted to the ICU at high risk for VTE, is effective in reducing the incidence of PE, and cost effective in terms of preventing fatal PE.
